# A novel mutation *KCNJ11* R136C caused *KCNJ11*-MODY

**DOI:** 10.1186/s13098-021-00708-6

**Published:** 2021-08-31

**Authors:** Yaning Chen, Xiaodong Hu, Jia Cui, Mingwei Zhao, Hebin Yao

**Affiliations:** Department of Endocrinology, Sixth Medical Center of PLA General Hospital, 6# Fucheng Road, Haidian District, Beijing, 100048 China

**Keywords:** *KCNJ11*-MODY, K_ATP_ channel, Diabetes mellitus

## Abstract

A young female patient, diagnosed with diabetes mellitus at the age of 28 years old in 2009, carries *KCNJ11* R136C by whole exome sequencing and her daughter doesn’t carry this mutation. Bioinformatics software predicted that the 136th amino acid is highly conservative and the mutation is deleterious. *KCNJ11* R136C can result in the change of channel port structure of K_ATP_ channel. So she was diagnosed as *KCNJ11*-MODY.

## Background

Maturity-onset diabetes of the young (MODY) is a group of clinically heterogeneous and hereditary diabetes, caused by a single-gene mutation involving in the development and function of β-cells [1]. According to the involved genes and clinical phenotypes, 14 subtypes of MODY have been reported [2]. *KCNJ11*-MODY (known as MODY13) is an autosomal dominant disorder caused by *KCNJ11* gene mutations, which codes inward rectifying potassium channel (Kir)6.2. Four Kir6.2 subunits and four sulphonylurea receptor 1 (SUR1) subunits constitute K_ATP_ channel. K_ATP_ channel plays an important role in insulin secretion. Here we report a young female patient was misdiagnosed with type 2 diabetes and carried *KCNJ11* p.R136C (c. 406C > T), a new mutation causing *KCNJ11*-MODY.

## Patient and methods

### Subjects

The informed consent was obtained from the participants (or the child, from her mother). The study complied with Declaration of Helsinki. Regrettably, the proband is an orphan and blood samples of her parents can’t be obtained.

In 2009, the proband was diagnosed with diabetes mellitus during pregnancy when she was 28 years old. Insulin treatment was prescribed, but blood glucose was controlled poor. Caesarean section was performed at 38 weeks of gestation because of a large fetus and the newborn weighed 5500 g. She had taken metformin 1500 mg/d and acarbose 150 mg/d since 2011. Fasting blood glucose (FBG) was about 12 mmol/L and postprandial 2 h blood glucose was 16 mmol/L or so. She was admitted to our department in April 2017. Her height, weight, waist circumference and BMI were 172 cm, 73 kg, 95 cm and 24.68 kg/m^2^, respectively. Glycosylated Hemoglobin was 10.3% and Anti-glutamic acid decarboxylase, anti-islet-cell, and anti-insulin antibodies were all negative. Her FBG, fasting C-peptide, insulin concentration were 13.06 mmol/L, 1.49 ng/ml and 15.06 mIU/L, respectively. She was diagnosed with type 2 diabetes, and 52 IU of glargine was added. However, FBG was still above 10 mmol/L. In September 2020, retinal examinations by an ophthalmologist, urinary albumin/creatinine and electrophysiological testing of peripheral neuropathy were assessed and all of them were normal.

### Whole exome sequencing (WES) and analysis

Peripheral blood samples (5 ml per individual) were drawn from the proband and her daughter. Genomic DNA was extracted according to the manufacture’s standard procedure (MagPure Buffy Coat DNA Midi KF Kit) and then single individual DNA library was built. The library was enriched 16–24 h(47 ℃) by array hybridization (Roche NimbleGen, Madison, USA), followed by elution and post-capture amplification. The products were then subjected to Agilent 2100 Bioanalyzer and BMG to estimate the magnitude of enrichment. The qualified products were pooled and quantified according different library quantities, then the single strand of library products were prepared for circularization and made DNB, finally, sequenced with PE100 + 100 on MGISEQ-2000.

To detect the potential variants, we performed bioinformatics processing and data analysis after receiving the primary sequencing data. We used previously published filtering criteria to generate “clean reads” for further analysis [3]. We used GATK software [4] to detect single-nucleotide variants (SNVs) and indels. All SNVs and indels were filtered and estimated via multiple databases, including NCBI dbSNP, HapMap, 1000 human genome dataset and database of 100 Chinese healthy adults.

### Sanger validation

Sanger sequencing was performed to confirm the variants detected with whole exome sequencing. Bidirectional sequencing of the purified PCR products was performed with a 3130 XL sequencer (Applied Biosys-tem, Foster City, USA).

### Functional prediction

To predict the effect of missense variants, we used dbNSFP [5], which contains seven well-established in silico prediction programs [Scale-Invariant Feature Transform (SIFT), Polyphen2, LRT, MutationTaster, and PhyloP]. Pathogenic variants are assessed under the protocol issued by the American College of Medical Genetics and Genomics [6]. The Human Gene Mutation Database was used to screen mutations reported in published studies.

## Results

### Mutation analysis

**Fig. 1:**
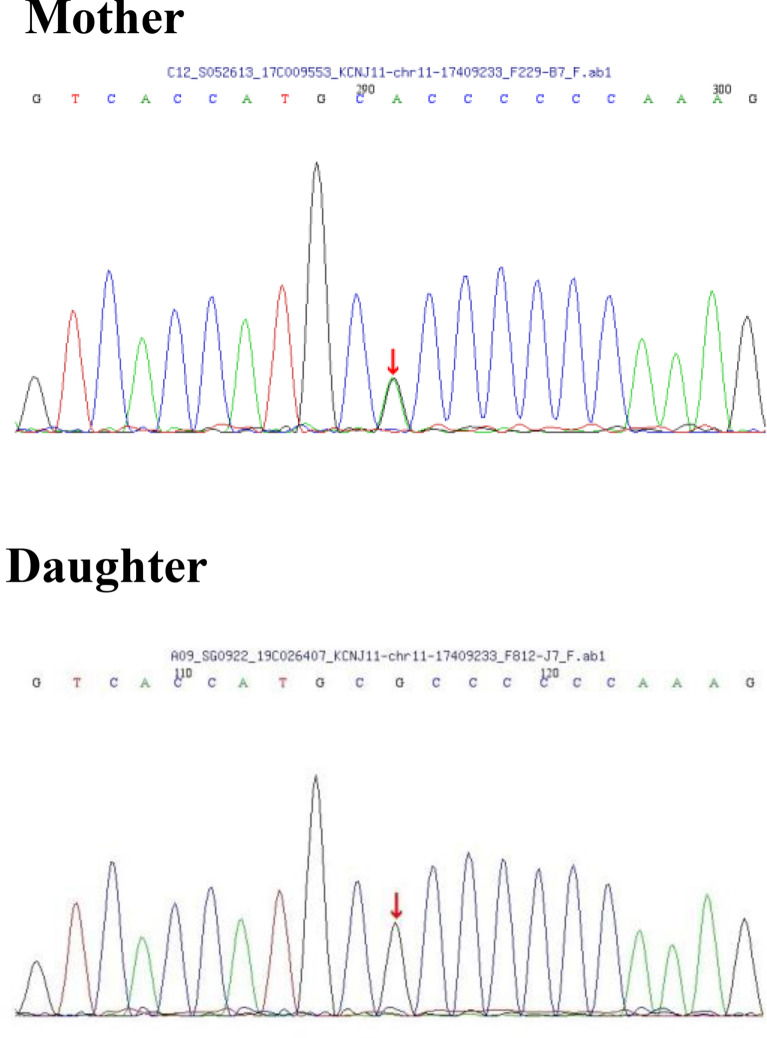
The genetic test report of the proband(mother) and her daughter

WES analysis was carried out in the proband and found she carried a single variant *KCNJ11* c.406C > T(p.R136C). Sanger Sequencing of *KCNJ11* c.406C > T gave a negative result in her daughter (see Fig. 1).

### Search for R136C mutation of *KCNJ11* gene

Referring to 1000 Genomes database, ESP6500si_all database, ExAC database and Genome Aggregation Database (GnomAD), the frequency of this mutation in public population databases is extremely low (ESP6500:-, 1000 Genomes:-, EXAC: 0.000008, GnomAD: 0.000004).

### Conservative property of R136C mutation in *KCNJ11* gene

**Fig. 2:**
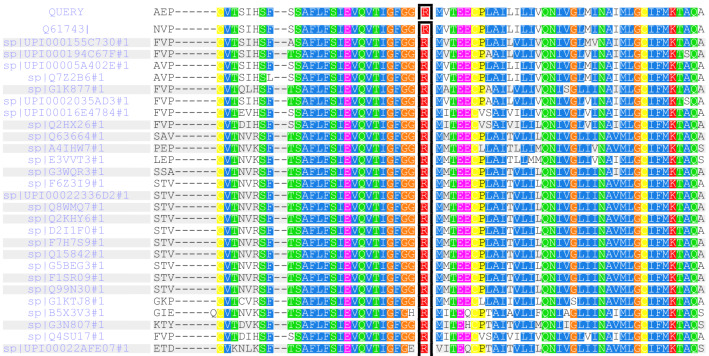
The 136th amino acids of Kir6.2 subunit in 28 different species are arginine

The mutation site of *KCNJ11* R136C was analyzed for conservative property among multiple species: Phylop: conservative, GERP++ _RS: conservative. In all 28 different species, the 136th amino acids of Kir6.2 subunit are arginine, which is highly conserved (see Fig. 2) and a functionally important residue.

### Prediction of pathogenicity of *KCNJ11* R136C

According to the criteria for classifying pathogenic variants of the American College of Medical Genetics, the variant was considered to be likely pathogenic, Pathogenic Moderate (PM)1 + PM2 + PM3 + PM5 + Pathogenic Supporting(PP)3. According to SIFT:D, Condel:deleterious, MutationTaster:D and Polyphen2:D, this mutation can result in changes in protein function. The results of SIFT and Polyphen-2 software showed that the score of SIFT was 0 and that of Polyphen-2 was 1.

### Structure prediction of Kir6.2 subunit and K_ATP_ channel carrying *KCNJ11* R136C

**Fig. 3:**
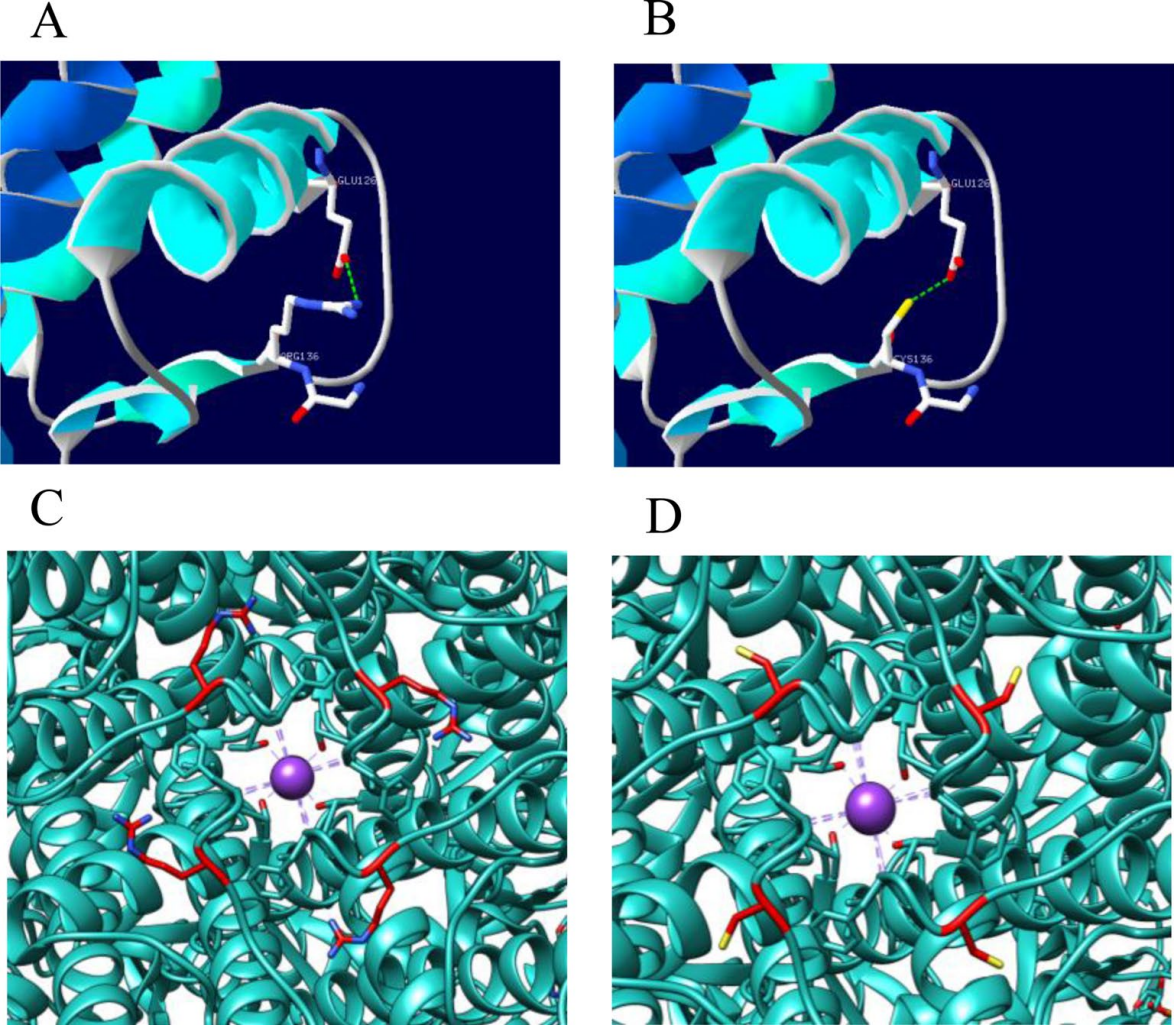
The real analytical structure of wide-type Kir6.2 subunit and K_ATP_ channel and Kir6.2 subunit and K_ATP_ channel carrying *KCNJ11* R136C predicted by computer software. **A** A hydrogen bond is formed between R136 and E126 in wide-type Kir6.2. **B** The hydrogen bond still exists between R136C and E126 in Kir6.2 subunit carrying *KCNJ11* R136C. And the real analytical structure predicted by computer software changes, marked by color yellow. **C** In wide-type K_ATP_ channel, color blue in the middle is potassium ions, which is in the channel port. **D** In K_ATP_ channel carrying *KCNJ11* R136C, the amino acid changes from hydrophilic basic amino acid with a positive charge to hydrophilic neutral amino acid, which can change the charge distribution of the channel port and thus affect the function of K_ATP_ channel

*KCNJ11* c.406C > T(p.Arg136Cys) changed the 136th amino acid from hydrophilic basic amino acid arginine with a positive charge to hydrophilic neutral amino acid cysteine, which can cause the charge distribution of the channel port. In wild-type K_ATP_ channel, Arg136 is located in a β turn at the port of the channel. So the mutation Arg136Cys may result in the change of β turn structure and further change the structure of pore portal. The real analytical structure of Kir6.2 subunit and K_ATP_ channel carrying *KCNJ11* R136C predicted by computer software is shown in Fig. 3.

## Discussion

This paper reports a young female patient who carried *KCNJ11* R136C mutation. She was considered as *KCNJ11*-MODY and prescribed by metformin, acarbose and glimepiride. Blood sugar control improved significantly.

*KCNJ11*-MODY is an autosomal dominant diabetes mellitus caused by mutations in *KCNJ11* gene, first reported by Bonnefond et al*.* in 2012. *KCNJ11* gene is located at 11p15.1, contains only one exon and encodes Kir6.2 subunit. Kir6.2 contains the binding sites of ATP and phosphatidylinositol 4,5-diphosphate, which can inhibit and activate K_ATP_ channel, respectively [7, 8]. K_ATP_ channel on pancreatic β cells couples energy metabolism and electrical activity and plays an important role in the process of insulin secretion [9]. Under sub-stimulus glucose concentration, the membrane potential of β cell is affected by the conductance of K_ATP_ channel, maintaining the cell member potential at a hyperpolarized level. When blood glucose rises, glucose is quickly taken up by β cell and metabolized into ATP. ATP binds to K_ATP_ channel, closing the channel, depolarizing cell membrane, opening voltage-gated calcium channels, calcium ion influx and triggering insulin vesicles release.

Mutations in *KCNJ11* gene affect the activity of K_ATP_ channels, causing abnormal insulin secretion in pancreatic β-cells. Activating mutation can cause a decrease in the affinity of ATP to the channel in pancreatic β cells. K_ATP_ channel can’t be closed normally under the stimulation of glucose and cell membrane continues to be in a hyperpolarized state. Extracellular Ca^2+^ can’t inflow and insulin can’t be secreted normally, which leads to a series of continuous and varying degrees of glucose metabolism abnormalities, including neonatal diabetes mellitus, impaired fasting glucose, impaired glucose tolerance and *KCNJ11*-MODY. Inactivation mutation in *KCNJ11* gene can lead to continuous closure of K_ATP_ channel, continuous depolarization of β cell membrane, continuous inflow of extracellular Ca^2+^, excessive secretion and release of insulin, resulting in congenital hyperinsulinism hypoglycemia.

Bonnefond et al. [10] reported among a four-generation family of thirty-seven members, twelve members carried *KCNJ11* E227K mutation: three members (aged 11–40 years old) with normal glucose metabolism, and nine members with abnormal glucose metabolism. They were diagnosed with diabetes at the age of 13–59 years old. This mutation can cause a decrease in the sensitivity of ATP to K_ATP_ channel. Before that, there were two reports about *KCNJ11* mutation causing diabetes. Some patients might also be diagnosed with *KCNJ11*-MODY. Four members in a three-generation Japanese family carried *KCNJ11* C42R and were diagnosed with diabetes, three cases of which were diagnosed at the age of 3, 22 and 26 years old. Functional identification showed that the mutant channel is less sensitive to ATP [11]. An Italian family carrying *KCNJ11* c.679G > C and c.680A > T(p.E227L) was reported, two of which can be considered as *KCNJ11*-MODY [12].

Ang et al*.* [13], Ren et al*.* [14], Li et al*.* [15] and He et al*.* [16] reported MODY13 family trees in Chinese, *KCNJ11* c.392 T > C (p.I137T)、c.679G > A(E227K) 、c.602G > A(p.R201H)and c.142A > G(p.N48D), but these authors didn’t testify the function of mutant channels. Liu et al*.* [17] reported three new *KCNJ11* heterozygous mutations in three MODY diabetic families: two activating mutations R27H and R192H, one inactivating mutation S116F117del. In vitro studies showed that the sensitivity of K_ATP_ channel to ATP carrying R27H or R129H is significantly reduced. The authors also pointed out that *KNCJ11* mutation was measured in 3.2% of 96 Chinese families with early-onset type 2 diabetes mellitus.

Most of the reported *KCNJ11*-MODY patients had successfully converted from insulin to sulfonylureas, which can not only improve blood glucose, reduce medical costs, but also improve the quality of life. The key point lies in the accurate screening and effective identification of *KCNJ11*-MODY.

It was reported that the mutation of *KCNJ11* R136 to other amino acids, such as Arg136His, Arg136Leu, can cause congenital hyperinsulinemia [18, 19], and the authors didn’t conduct functional studies on related mutations. More surprisingly, Bellann ´e-Chantelot et al*.* [20] reported that an infant with congenital hyperinsulinemia carried *KCNJ11* R136C mutation. Park et al*.* [21] also reported a Korean infant with congenital hyperinsulinemia carried Arg136Cys and Ala187Val compound heterozygous mutations, but the authors didn’t describe the above two cases in detail. The function of the mutant channel wasn’t been studied. The same mutation can cause different clinical phenotype, which also reflects the clinical heterogeneity of *KCNJ11* gene mutation.

Further studies are required to carry out the functional identification and related research of *KCNJ11* R136C to gain a deeper understanding of the clinical heterogeneity.
